# The Effect of Test Method on Visual Acuity in School Children Aged 4–5

**DOI:** 10.22599/bioj.262

**Published:** 2022-04-05

**Authors:** Rebecca Lewis, Charlotte Codina, Helen Griffiths

**Affiliations:** 1 Leeds Teaching Hospitals, GB; 2 The University Of Sheffield, GB; 3 Sheffield Children’s NHS Foundation Trust, GB

**Keywords:** Children’s visual acuity, vision test method, crowded logMAR

## Abstract

**Purpose:**

This study compared two different methods of testing visual acuity (VA) in children aged 4–5 years (The UK’s school vision screening target age). A conventional vision test method was compared to a reversed presentation order of logMAR, where letters are presented in ascending size order up to vision threshold. Threshold VA, test duration and concentration were compared, to assess the most accurate and efficient method of VA testing in this age group, to determine the most clinically and cost-effective method for vision screening.

**Methods:**

Thirty-four participants completed the study (15 males, 19 females, age range 53–65 months, mean age 59 months’ ±3.7 months). VA was measured in logMAR. Keeler Crowded logMAR screening plates determined the starting line on the vision chart to ensure the initial optotype size was either seen or not seen for the conventional and reversed test methods respectively. Test duration was measured in seconds and a concentration score was given by the examiner.

**Results:**

The median VA was 0.17 logMAR for each test method. There was no significant difference in the VA outcomes between each test method (p = 0.46). The reversed method was significantly quicker to complete, with a median reduction in test duration of 28 seconds (p = 0.002). There was no difference in concentration levels between the test methods.

**Conclusion:**

Both test methods gave the same VA threshold, and are therefore comparable. The reversed method was significantly quicker to complete which could benefit school vision screening services and busy clinical contexts.

## Introduction

School vision screening programmes are recommended for children aged 4–5 years (PHE 2017) and for the majority of children in the UK, it is their first vision test (Bruce et al. 2018). Public Health England (PHE) recommends in national vision screening guidelines, referral for diagnostic assessment if VA is worse than 0.200 logMAR in either eye on the Keeler crowded logMAR test (UK NSC 2019). However, as vision screening in England is not a national programme, there is national variability of how screening is performed, as well as a variation throughout Europe (EU Screen 2020).

The testability of VA improves with age, most dramatically in the first 24 months of life, followed by consistent slower improvement in children (Leone et al. 2014), due to reduced test compliance and cognitive ability (Solebo, Cumberland & Rahi 2015). Maturation of line acuity is still occurring between the ages of 4–9 (Norgett & Siderov 2011), yet amblyopia treatment has greater success before age 7 (Gunton 2013). Younger children are more likely to fail vision screening even with the use of age-appropriate tests and matching cards (Griffiths, Carlton & Mazzone 2019). Confounding factors of vision testing in young children include the wellbeing of the child and their concentration, environmental (such as distracting factors in the room), and methodological (the method employed by the examiner) (Anstice & Thompson 2014). Glasgow acuity cards (McGraw et al. 2000), now more commonly known as Keeler Crowded logMAR test (KCLT), is a frequently used crowded vision test in UK practice (Anstice et al. 2017), designed using the ETDRS chart principles (Bailey & Lovie-Kitchin 1976) for children aged 3–6 years (McGraw et al. 2000). VA however is now increasingly being assessed on computer-based displays that also incorporate the ETDRS chart principles.

Presenting letters in a reversed presentation order, from smallest to largest has been suggested by Thomson and Evans (1999) as an alternative test method for vision screening, to reduce test duration and consequently improve cost-effectiveness. Reduced testing time could better maintain concentration and therefore could increase the accuracy of the vision threshold. Conventionally, VA testing commences with a visible optotype and progresses in a descending size order to vision threshold. A reversed presentation order commences the test with a small optotype size that may not be seen and progresses in an ascending size order to threshold. Test duration may not be reduced with the reversed method where subnormal vision is present; the reversed method is based on most children achieving normative VA thresholds (Griffiths, Carlton & Mazzone 2018). Reversed presentation of non-seeing to seeing could elongate test duration and affect the accuracy of the threshold VA. The reversed presentation method could reduce concentration when commencing with an unidentifiable optotype, possibly due to altered confidence or attention on the task. Mai et al. (2011) reported that children’s brains at age 4–5 appear to be more responsive to positive feedback, with positive feedback increasing task motivation.

The aim of this study was to compare the traditional and reversed methods of VA testing in children aged 4–5 years using a computerised crowded logMAR test, to measure and analyse potential differences in VA, test duration and concentration.

## Methods

Ethical approval was obtained from the HRA prior to the study commencing (reference 19/LO/1631). Thirty-four participants (15 males, 19 females) age ranges 53–65 months (mean age 59 months SD ±3.7) were recruited within a two month period. The sample size was based on a repeated measures power calculation (effect size of 0.5, alpha error value 0.05, and power 0.8, G*Power software). All participants were registered patients of a single hospital eye service and were in reception year of a mainstream school (age 4–5). Participant parents/guardians were contacted by telephone to explain the study to prepare them at least 24 hours before their appointment for potential participation. Further written information was given on arrival for the appointment and consent forms were signed if willing to participate.

Nineteen participants were aged between 53–59 months, and 15 participants were aged 60–65 months. Twenty-six of the 34 (76%) participants were follow-up patients and 8 (24%) were new patients. For 23 (68%) participants, this study was their first experience with a crowded logMAR VA test. The orders of test methods were counterbalanced. Nine right eyes and 23 left eyes were randomly selected by a random number generator whilst the other eye was occluded. Spectacles were permitted if worn or unaided where none prescribed. Data collection took place in the same clinic room with one examiner (RB). A letter matching key card was offered to all participants, to increase the testability of this age group (Rydberg et al. 1999).

KCLT screening plates (Keeler, UK) were used prior to each test method to determine the initial logMAR size, as well as introduce participants to the test methods. The conventional test method was commenced two lines above the screening result and the reversed test method was commenced two lines below the screening result, to ensure each method was commenced with an identifiable or unidentifiable letter respectively.

The Test Chart 2000 Xpert displayed crowded logMAR with the same spacing as KCLT, and a screen luminance of 150 cdm^–2^, calibrated for a 4-metre testing distance. Participants were asked to identify all optotypes on a presented line. If an incorrect response was given, one further attempt was permitted and the letter was not tested again if two incorrect responses were given. If the child did not want to attempt the letter, no repeated instruction was given and the examiner moved onto the next letter. Optotype size was changed consecutively in 0.100 logMAR units in the appropriate direction for each method. The conventional test method followed the termination rule described by McGraw et al. (2000), when three or more letters on a logMAR line were not correctly identified, the test was terminated and the result calculated. The reversed test method was terminated when the participant was able to identify at least three letters on a crowded logMAR line.

The test duration for each method was measured in seconds using a stopwatch. The level of concentration was scored by the examiner based on a modified version of the Child Concentration Inventory (CCI) from Becker et al. (2015). The CCI provides information on concentration deficits and symptoms of reduced attention in children, used originally to identify a subset of children with attention-deficit/hyperactivity disorder (ADHD) who showed clinical levels of inattentive symptoms but few, if any symptoms of hyperactivity/impulsivity. It has not been previously used in VA assessments; there is no validated scoring model for this. The CCI usually provides a concentration score between 0–9 based on three categories; slow, sleepy and daydreaming (Becker et al. 2015) where 0 = ‘not at all’ and 3 = ‘very’. A modified approach was taken excluding the category ‘sleepy’ as this was not relevant to the current study, thus allowing a total score between 0–6 to be given by the examiner (***Table 1***).

**Table 1:** Concentration scoring using the modified Child Concentration Inventory (CCI) (Becker et al. 2015).


	0 = NOT AT ALL 1 = SLIGHTLY 2 = MODERATELY 3 = VERY

Slow/unmotivated	_ / 3

Daydreaming	_ / 3

Overall concentration score	_ / 6


## Results

Thirty-four participants completed the study. ***Table 2*** shows the visual diagnoses of the 34 participants. Thirty-two eyes (94%) were not amblyopic. Twenty-three of the 34 participants (68%) wore refractive correction for the VA measurement. Nineteen of the 34 participants (56%) opted to use the key card to match letters for both test conditions. Visual acuity and test duration data was not normally distributed for the reversed method as shown by the Shapiro-Wilk test therefore non-parametric tests (Wilcoxon signed rank test) were used in statistical analysis.

**Table 2:** Diagnoses of participants (N = 34). Total > 34 as some participants had combined diagnoses.


DIAGNOSIS	FREQUENCY/34 (%)

Hypermetropia	13 (38%)

Astigmatism	12 (35%)

Myopia	6 (18%)

Constant strabismus	8 (24%)

Intermittent/latent strabismus	7 (21%)

Microtropia	1 (3%)

Strabismic amblyopia	7 (21%)

Anisometropic amblyopia	3 (9%)

Mixed amblyopia	1 (3%)

Ocular pathology	4 (12%)

Cranial pathology	3 (9%)

No apparent defect	1 (3%)


### Visual acuity

The median VA was the same for each test method, 0.17 logMAR (***Table 3***). The ranges of VA are also shown in ***Table 3*** and are similar for both test methods. Twenty-five participants (74%) achieved normative VA of 0.200 logMAR or better on at least one of the test methods, whereas only 17 participants (50%) achieved normal vision for both test methods. There were four participants (12%) that achieved normative VA for the test method performed first and were below normative VA for the test method performed second, irrespective of the method order used. 2/34 participants had ≥1 logMAR line reduction in VA outcome for the test method performed second.

**Table 3:** Descriptive statistics of VA.


	CONVENTIONAL TEST ORDER (LOGMAR) N = 34	REVERSED TEST ORDER (LOGMAR) N = 34

Median with confidence limits (approx 95% confidence)	0.17 (0.12 to 0.24)	0.17 (0.12 to 0.22)

Interquartile range	0.18	0.13

Range	–0.04 to 0.52	–0.06 to 0.56


**Figure 1:**
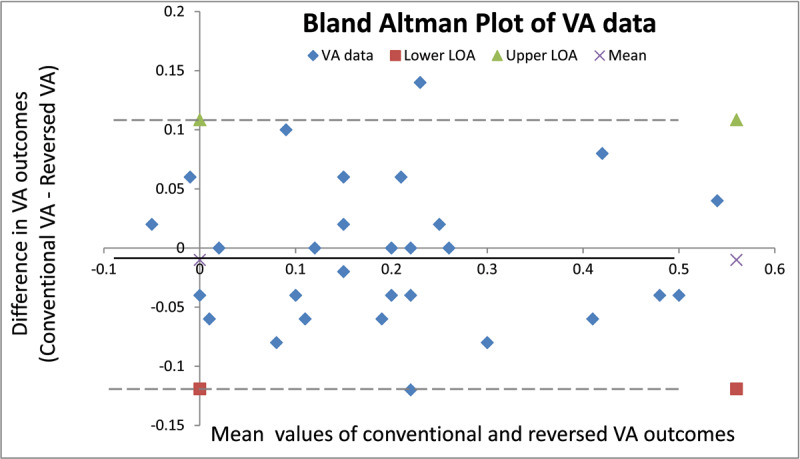
Bland Altman Plot of VA test method. The difference between the VA outcomes was calculated by subtracting the reversed method outcome from the conventional method outcome. The middle solid line represents the mean bias. The upper and lower 95% limits of agreement are represented by the dashed lines. N = 34.

The Wilcoxon signed rank test showed that there was no significant difference in VA recorded with the conventional or reversed method (Z = –0.747, p = 0.46). Spearman’s rank correlation showed a significant positive linear relationship between the conventional and reversed VA measures (r = 0.88, p < 0.001). VA from each method was tested for agreement using a Bland-Altman plot (***Figure 1***). ***Figure 1*** shows that the bias is clinically small between methods. The limits of agreement are narrow and consistent across all acuity levels, showing that the two methods are essentially equivalent. Most participants had ≤1 logMAR line of difference in the VA outcome of each test method (32/34 participants), and the majority of participants had an average VA within normal limits (20/34 participants).

### Test duration

The test duration of the reversed method (median 64 secs) was significantly less (28 seconds) than the conventional method (median 94 secs) as shown in ***Table 4*** with the large range in seconds for both test methods. Twenty-six of the 34 participants (77%) achieved shorter test duration on the reversed method. Wilcoxon signed rank test shows the reversed test method was significantly quicker for participants to complete (Z = –3.09, p = 0.002).

**Table 4:** Descriptive statistics of test duration data.


	CONVENTIONAL ORDER TEST DURATION (SEC)	REVERSED ORDER TEST DURATION (SEC)

Median with confidence limits of the median (approximately 95% confidence)	92 (74–107)	64 (55–80)

Interquartile Range	52.25	38.75

Range (participants with normal VA)	42–162	28–175

Range (participants with subnormal VA)	65–178	32–130


There was no significant difference in test duration between younger participants N = 19 (age 53–59 months) and older participants N = 15 (aged 60–65 months). There was no significant difference in test duration between experienced (N = 26) or new patient participants N = 8, although these subgroups are small for analysis.

### Concentration

**Figure 2:**
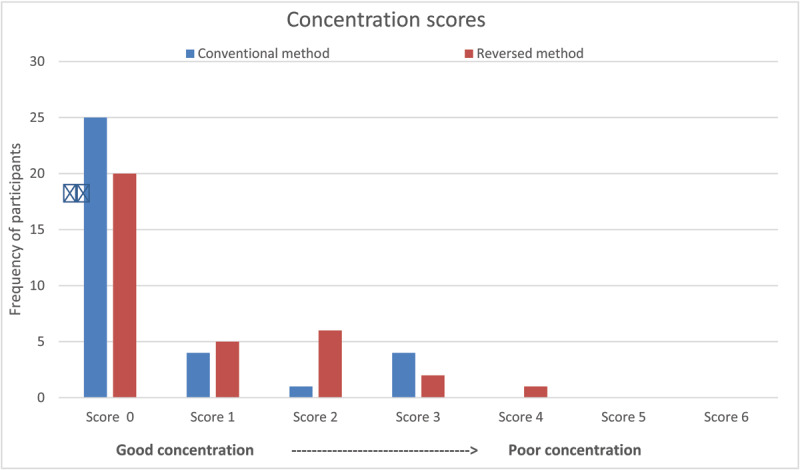
Concentration scores for each test method.

The concentration scores for both test methods are shown in ***Figure 2***. The mode concentration scores for both test methods was zero; indicating a high level of concentration under each test condition: half the participants achieved a concentration score of 0 for both methods. The two participants who had a VA outcome reduced by >1 logMAR line for the method performed second also had a worse concentration score for the second test method compared to their first. The poorest concentration score given was 4/6, which was a combination of daydreaming and slowness on the reversed method. The same participant achieved 0/6 for the conventional method performed first demonstrating a loss in concentration due to repetition of tests. A higher percentage of children (74%) achieved an excellent concentration score of 0 for the conventional method, compared to the reversed method (59%), however the difference was not significant (Wilcoxon signed rank test, W = 134, critical value = 182). Only 9/34 of participants had VA below the normative level on both test methods. Further statistical analysis on their concentration was not reviewed.

## Discussion

Essentially, conventional and reversed method VA testing produced equivalent results (***Table 3***) in this sample of 34 children routinely attending the eye clinic. Most participants (77%) had shorter test duration for the reversed test method, with a median reduction of 28 seconds, showing it was a more efficient method for finding threshold VA, after the use of screening plates. The reduction in test duration would allow for more children to be tested per session in a screening programme, which would increase cost-effectiveness, and this study suggests concentration would not be affected.

Convenience sampling was used due to the availability of these participants during their already scheduled Orthoptic appointments. The use of a random number generator resulted in an equal chance of testing an eye with normal vision in a participant with a unilateral defect. It had been predicted a wider range of VA would be recruited from a hospital eye service in comparison to a general population of children aged 4–5. Most eyes tested in this study had normative VA, limiting the generalisation of these results for children aged 4–5 with subnormal VA. Children who have subnormal VA would take longer to reach threshold VA when using a reversed test method without the use of screening plates. Further research is therefore required for children with subnormal VA and the reversed test method.

The results of this study are applicable to typically developing children age 4–5, due to the exclusion of diagnosed attention deficits. Although there was no significant difference in concentration for each test method, this conclusion may be hindered by the scoring method which was adapted from its originally validated use, and examiner subjectivity. To the author’s knowledge, no other research has been based on the concentration of a child during a vision assessment, even though it is understood a child’s concentration is related to their performance during a vision test and is also affected by the age of the child, more so when under age 6 (Leone et al. 2014). A more specifically designed concentration scoring system is required for increased sensitivity of assessing concentration during a vision assessment and its relationship to threshold VA and test duration.

The age range recruited was narrow and specific to children in reception year at the time of their participation in the study, which is the same age group that would undergo a recommended school screening eye test (UK NSC 2019). Data collection took place between January–March 2020, approximately half way through the academic year. If data collection had been performed earlier or later in the academic year, results may have differed due to the children having a different level of experience with capital letters, and different concentration dependent on age at the time the study was undertaken (Leone et al. 2014). The use of a key card significantly increased testability in this age group, as 56% required it to do the logMAR test. There was an increased chance of guessing with the key card, but the participant acted as their own control and had the key card for both test methods.

Thomson and Evans (1999) reported average test duration of 180 seconds to complete a VA assessment of both eyes using the reversed method. By adding the average test durations of each test method in this study for an approximation of test duration for two eyes, there was average time of 167 seconds, similar to the findings of Thomson and Evans (1999). However, the range of test durations is larger than what is previously reported by School Screener© (2019), of an average of 60 seconds test duration for children with normal vision, and 120 seconds for children with subnormal vision. The range of test durations for participants with normal VA and subnormal VA on each test method is shown in ***Table 4***. The large range and variability are likely representative of the young age group in this study; adults would likely have smaller test durations with less variation (Anstice et al. 2017). The standard deviation was large and CIs of the median (to give an approximate 95% confidence) were wide, owing to the small sample size and large variation. Due to this variation it is difficult to conclude an expected test duration in a general population of children aged 4–5 from this study. Older participants (60–65 months) were less likely to have a difference in the test duration for each method, however there was no statistical significance therefore nothing can be inferred about the test duration data from this narrow age range included. The outlier test duration of 175 seconds recorded for the reversed test method may not be a true outlier of a general population. Further research would be required with a larger sample size.

Counterbalancing was important to protect results from fatigue. The 12% (4 participants) that achieved less than normative VA for one method and not the other is presumed to be related to the repeatability of VA testing in this young age group, and potentially could still happen even if the same test method had been repeated twice. Keeler Crowded logMAR screening plates assumed close approximation of VA threshold. It was also assumed those with a concentration score of 0 completed the vision assessment to the best of their ability, with the outcome VA being truly representative of their threshold VA. Although inter-examiner variability was prevented by only having one examiner, the examiner was not blinded to any previously recorded VA’s. Attempts were made to reduce examiner bias by having specific test termination and starting criteria.

## Conclusion

This study has shown that testing using the conventional method and reversed method give essentially equivalent visual acuity thresholds, in children age 4–5 with relatively normal visual acuity. However, the reversed method is significantly quicker and this method may be of benefit to vision screening programmes, wherein the collective time-saving from multiple tests may be more economical, as well as in busy clinical settings to improve efficiency.

## Additional File

The additional file for this article can be found as follows:

10.22599/bioj.262.s1 The Effect of Test Method on Visual Acuity in School Children Aged 4–5. A comparative study of two different methods of testing visual acuity in children aged 4–5 years.
